# Supplementary material for: Resolving the full spectrum of human genome variation using Linked-Reads
Source: Genome Res. 2019 Apr;29(4):635–45. doi: 10.1101/gr.234443.118 (PMC6442396; doi:10.1101/gr.234443.118)
Supplement: Supplemental Material [file supp_gr.234443.118_Supplemental_longranger-2.2.zip › longranger-2.2.tar.bz2!/lr2.2-public/lib/go/src/code.google.com/vitess/third_party/py/mock-1.0.1.tar.gz!/mock-1.0.1/html/examples.html]

Further Examples — Mock 1.0.1 documentation


### Navigation

- index
- next |
- previous |
- Mock 1.0.1 documentation »

# Further Examples¶

For comprehensive examples, see the unit tests included in the full source
distribution.

Here are some more examples for some slightly more advanced scenarios than in
the *getting started* guide.

## Mocking chained calls¶

Mocking chained calls is actually straightforward with mock once you
understand the return\_value attribute. When a mock is called for
the first time, or you fetch its return\_value before it has been called, a
new Mock is created.

This means that you can see how the object returned from a call to a mocked
object has been used by interrogating the return\_value mock:

```
>>> mock = Mock()
>>> mock().foo(a=2, b=3)
<Mock name='mock().foo()' id='...'>
>>> mock.return_value.foo.assert_called_with(a=2, b=3)
```

From here it is a simple step to configure and then make assertions about
chained calls. Of course another alternative is writing your code in a more
testable way in the first place...

So, suppose we have some code that looks a little bit like this:

```
>>> class Something(object):
...     def __init__(self):
...         self.backend = BackendProvider()
...     def method(self):
...         response = self.backend.get_endpoint('foobar').create_call('spam', 'eggs').start_call()
...         # more code
```

Assuming that BackendProvider is already well tested, how do we test
method()? Specifically, we want to test that the code section # more
code uses the response object in the correct way.

As this chain of calls is made from an instance attribute we can monkey patch
the backend attribute on a Something instance. In this particular case
we are only interested in the return value from the final call to
start\_call so we don’t have much configuration to do. Let’s assume the
object it returns is ‘file-like’, so we’ll ensure that our response object
uses the builtin file as its spec.

To do this we create a mock instance as our mock backend and create a mock
response object for it. To set the response as the return value for that final
start\_call we could do this:

> mock\_backend.get\_endpoint.return\_value.create\_call.return\_value.start\_call.return\_value = mock\_response.

We can do that in a slightly nicer way using the configure\_mock()
method to directly set the return value for us:

```
>>> something = Something()
>>> mock_response = Mock(spec=file)
>>> mock_backend = Mock()
>>> config = {'get_endpoint.return_value.create_call.return_value.start_call.return_value': mock_response}
>>> mock_backend.configure_mock(**config)
```

With these we monkey patch the “mock backend” in place and can make the real
call:

```
>>> something.backend = mock_backend
>>> something.method()
```

Using mock\_calls we can check the chained call with a single
assert. A chained call is several calls in one line of code, so there will be
several entries in mock\_calls. We can use call.call\_list() to create
this list of calls for us:

```
>>> chained = call.get_endpoint('foobar').create_call('spam', 'eggs').start_call()
>>> call_list = chained.call_list()
>>> assert mock_backend.mock_calls == call_list
```

## Partial mocking¶

In some tests I wanted to mock out a call to datetime.date.today() to return
a known date, but I didn’t want to prevent the code under test from
creating new date objects. Unfortunately datetime.date is written in C, and
so I couldn’t just monkey-patch out the static date.today method.

I found a simple way of doing this that involved effectively wrapping the date
class with a mock, but passing through calls to the constructor to the real
class (and returning real instances).

The patch decorator is used here to
mock out the date class in the module under test. The side\_effect
attribute on the mock date class is then set to a lambda function that returns
a real date. When the mock date class is called a real date will be
constructed and returned by side\_effect.

```
>>> from datetime import date
>>> with patch('mymodule.date') as mock_date:
...     mock_date.today.return_value = date(2010, 10, 8)
...     mock_date.side_effect = lambda *args, **kw: date(*args, **kw)
...
...     assert mymodule.date.today() == date(2010, 10, 8)
...     assert mymodule.date(2009, 6, 8) == date(2009, 6, 8)
...
```

Note that we don’t patch datetime.date globally, we patch date in the
module that *uses* it. See *where to patch*.

When date.today() is called a known date is returned, but calls to the
date(...) constructor still return normal dates. Without this you can find
yourself having to calculate an expected result using exactly the same
algorithm as the code under test, which is a classic testing anti-pattern.

Calls to the date constructor are recorded in the mock\_date attributes
(call\_count and friends) which may also be useful for your tests.

An alternative way of dealing with mocking dates, or other builtin classes,
is discussed in this blog entry.

## Mocking a Generator Method¶

A Python generator is a function or method that uses the yield statement to
return a series of values when iterated over [1].

A generator method / function is called to return the generator object. It is
the generator object that is then iterated over. The protocol method for
iteration is \_\_iter\_\_, so we can
mock this using a MagicMock.

Here’s an example class with an “iter” method implemented as a generator:

```
>>> class Foo(object):
...     def iter(self):
...         for i in [1, 2, 3]:
...             yield i
...
>>> foo = Foo()
>>> list(foo.iter())
[1, 2, 3]
```

How would we mock this class, and in particular its “iter” method?

To configure the values returned from the iteration (implicit in the call to
list), we need to configure the object returned by the call to foo.iter().

```
>>> mock_foo = MagicMock()
>>> mock_foo.iter.return_value = iter([1, 2, 3])
>>> list(mock_foo.iter())
[1, 2, 3]
```

|  |  |
| --- | --- |
| [1] | There are also generator expressions and more advanced uses of generators, but we aren’t concerned about them here. A very good introduction to generators and how powerful they are is: Generator Tricks for Systems Programmers. |

## Applying the same patch to every test method¶

If you want several patches in place for multiple test methods the obvious way
is to apply the patch decorators to every method. This can feel like unnecessary
repetition. For Python 2.6 or more recent you can use patch (in all its
various forms) as a class decorator. This applies the patches to all test
methods on the class. A test method is identified by methods whose names start
with test:

```
>>> @patch('mymodule.SomeClass')
... class MyTest(TestCase):
...
...     def test_one(self, MockSomeClass):
...         self.assertTrue(mymodule.SomeClass is MockSomeClass)
...
...     def test_two(self, MockSomeClass):
...         self.assertTrue(mymodule.SomeClass is MockSomeClass)
...
...     def not_a_test(self):
...         return 'something'
...
>>> MyTest('test_one').test_one()
>>> MyTest('test_two').test_two()
>>> MyTest('test_two').not_a_test()
'something'
```

An alternative way of managing patches is to use the *patch methods: start and stop*.
These allow you to move the patching into your setUp and tearDown methods.

```
>>> class MyTest(TestCase):
...     def setUp(self):
...         self.patcher = patch('mymodule.foo')
...         self.mock_foo = self.patcher.start()
...
...     def test_foo(self):
...         self.assertTrue(mymodule.foo is self.mock_foo)
...
...     def tearDown(self):
...         self.patcher.stop()
...
>>> MyTest('test_foo').run()
```

If you use this technique you must ensure that the patching is “undone” by
calling stop. This can be fiddlier than you might think, because if an
exception is raised in the setUp then tearDown is not called. unittest2 cleanup functions make this simpler:

```
>>> class MyTest(TestCase):
...     def setUp(self):
...         patcher = patch('mymodule.foo')
...         self.addCleanup(patcher.stop)
...         self.mock_foo = patcher.start()
...
...     def test_foo(self):
...         self.assertTrue(mymodule.foo is self.mock_foo)
...
>>> MyTest('test_foo').run()
```

## Mocking Unbound Methods¶

Whilst writing tests today I needed to patch an *unbound method* (patching the
method on the class rather than on the instance). I needed self to be passed
in as the first argument because I want to make asserts about which objects
were calling this particular method. The issue is that you can’t patch with a
mock for this, because if you replace an unbound method with a mock it doesn’t
become a bound method when fetched from the instance, and so it doesn’t get
self passed in. The workaround is to patch the unbound method with a real
function instead. The patch() decorator makes it so simple to
patch out methods with a mock that having to create a real function becomes a
nuisance.

If you pass autospec=True to patch then it does the patching with a
*real* function object. This function object has the same signature as the one
it is replacing, but delegates to a mock under the hood. You still get your
mock auto-created in exactly the same way as before. What it means though, is
that if you use it to patch out an unbound method on a class the mocked
function will be turned into a bound method if it is fetched from an instance.
It will have self passed in as the first argument, which is exactly what I
wanted:

```
>>> class Foo(object):
...   def foo(self):
...     pass
...
>>> with patch.object(Foo, 'foo', autospec=True) as mock_foo:
...   mock_foo.return_value = 'foo'
...   foo = Foo()
...   foo.foo()
...
'foo'
>>> mock_foo.assert_called_once_with(foo)
```

If we don’t use autospec=True then the unbound method is patched out
with a Mock instance instead, and isn’t called with self.

## Checking multiple calls with mock¶

mock has a nice API for making assertions about how your mock objects are used.

```
>>> mock = Mock()
>>> mock.foo_bar.return_value = None
>>> mock.foo_bar('baz', spam='eggs')
>>> mock.foo_bar.assert_called_with('baz', spam='eggs')
```

If your mock is only being called once you can use the
assert\_called\_once\_with() method that also asserts that the
call\_count is one.

```
>>> mock.foo_bar.assert_called_once_with('baz', spam='eggs')
>>> mock.foo_bar()
>>> mock.foo_bar.assert_called_once_with('baz', spam='eggs')
Traceback (most recent call last):
    ...
AssertionError: Expected to be called once. Called 2 times.
```

Both assert\_called\_with and assert\_called\_once\_with make assertions about
the *most recent* call. If your mock is going to be called several times, and
you want to make assertions about *all* those calls you can use
call\_args\_list:

```
>>> mock = Mock(return_value=None)
>>> mock(1, 2, 3)
>>> mock(4, 5, 6)
>>> mock()
>>> mock.call_args_list
[call(1, 2, 3), call(4, 5, 6), call()]
```

The call helper makes it easy to make assertions about these calls. You
can build up a list of expected calls and compare it to call\_args\_list. This
looks remarkably similar to the repr of the call\_args\_list:

```
>>> expected = [call(1, 2, 3), call(4, 5, 6), call()]
>>> mock.call_args_list == expected
True
```

## Coping with mutable arguments¶

Another situation is rare, but can bite you, is when your mock is called with
mutable arguments. call\_args and call\_args\_list store *references* to the
arguments. If the arguments are mutated by the code under test then you can no
longer make assertions about what the values were when the mock was called.

Here’s some example code that shows the problem. Imagine the following functions
defined in ‘mymodule’:

```
def frob(val):
    pass

def grob(val):
    "First frob and then clear val"
    frob(val)
    val.clear()
```

When we try to test that grob calls frob with the correct argument look
what happens:

```
>>> with patch('mymodule.frob') as mock_frob:
...     val = set([6])
...     mymodule.grob(val)
...
>>> val
set([])
>>> mock_frob.assert_called_with(set([6]))
Traceback (most recent call last):
    ...
AssertionError: Expected: ((set([6]),), {})
Called with: ((set([]),), {})
```

One possibility would be for mock to copy the arguments you pass in. This
could then cause problems if you do assertions that rely on object identity
for equality.

Here’s one solution that uses the side\_effect
functionality. If you provide a side\_effect function for a mock then
side\_effect will be called with the same args as the mock. This gives us an
opportunity to copy the arguments and store them for later assertions. In this
example I’m using *another* mock to store the arguments so that I can use the
mock methods for doing the assertion. Again a helper function sets this up for
me.

```
>>> from copy import deepcopy
>>> from mock import Mock, patch, DEFAULT
>>> def copy_call_args(mock):
...     new_mock = Mock()
...     def side_effect(*args, **kwargs):
...         args = deepcopy(args)
...         kwargs = deepcopy(kwargs)
...         new_mock(*args, **kwargs)
...         return DEFAULT
...     mock.side_effect = side_effect
...     return new_mock
...
>>> with patch('mymodule.frob') as mock_frob:
...     new_mock = copy_call_args(mock_frob)
...     val = set([6])
...     mymodule.grob(val)
...
>>> new_mock.assert_called_with(set([6]))
>>> new_mock.call_args
call(set([6]))
```

copy\_call\_args is called with the mock that will be called. It returns a new
mock that we do the assertion on. The side\_effect function makes a copy of
the args and calls our new\_mock with the copy.

Note

If your mock is only going to be used once there is an easier way of
checking arguments at the point they are called. You can simply do the
checking inside a side\_effect function.

```
>>> def side_effect(arg):
...     assert arg == set([6])
...
>>> mock = Mock(side_effect=side_effect)
>>> mock(set([6]))
>>> mock(set())
Traceback (most recent call last):
    ...
AssertionError
```

An alternative approach is to create a subclass of Mock or MagicMock that
copies (using copy.deepcopy) the arguments.
Here’s an example implementation:

```
>>> from copy import deepcopy
>>> class CopyingMock(MagicMock):
...     def __call__(self, *args, **kwargs):
...         args = deepcopy(args)
...         kwargs = deepcopy(kwargs)
...         return super(CopyingMock, self).__call__(*args, **kwargs)
...
>>> c = CopyingMock(return_value=None)
>>> arg = set()
>>> c(arg)
>>> arg.add(1)
>>> c.assert_called_with(set())
>>> c.assert_called_with(arg)
Traceback (most recent call last):
    ...
AssertionError: Expected call: mock(set([1]))
Actual call: mock(set([]))
>>> c.foo
<CopyingMock name='mock.foo' id='...'>
```

When you subclass Mock or MagicMock all dynamically created attributes,
and the return\_value will use your subclass automatically. That means all
children of a CopyingMock will also have the type CopyingMock.

## Raising exceptions on attribute access¶

You can use PropertyMock to mimic the behaviour of properties. This
includes raising exceptions when an attribute is accessed.

Here’s an example raising a ValueError when the ‘foo’ attribute is accessed:

```
>>> m = MagicMock()
>>> p = PropertyMock(side_effect=ValueError)
>>> type(m).foo = p
>>> m.foo
Traceback (most recent call last):
....
ValueError
```

Because every mock object has its own type, a new subclass of whichever mock
class you’re using, all mock objects are isolated from each other. You can
safely attach properties (or other descriptors or whatever you want in fact)
to type(mock) without affecting other mock objects.

## Multiple calls with different effects¶

Note

In mock 1.0 the handling of iterable side\_effect was changed. Any
exceptions in the iterable will be raised instead of returned.

Handling code that needs to behave differently on subsequent calls during the
test can be tricky. For example you may have a function that needs to raise
an exception the first time it is called but returns a response on the second
call (testing retry behaviour).

One approach is to use a side\_effect function that replaces itself. The
first time it is called the side\_effect sets a new side\_effect that will
be used for the second call. It then raises an exception:

```
>>> def side_effect(*args):
...   def second_call(*args):
...     return 'response'
...   mock.side_effect = second_call
...   raise Exception('boom')
...
>>> mock = Mock(side_effect=side_effect)
>>> mock('first')
Traceback (most recent call last):
    ...
Exception: boom
>>> mock('second')
'response'
>>> mock.assert_called_with('second')
```

Another perfectly valid way would be to pop return values from a list. If the
return value is an exception, raise it instead of returning it:

```
>>> returns = [Exception('boom'), 'response']
>>> def side_effect(*args):
...   result = returns.pop(0)
...   if isinstance(result, Exception):
...     raise result
...   return result
...
>>> mock = Mock(side_effect=side_effect)
>>> mock('first')
Traceback (most recent call last):
    ...
Exception: boom
>>> mock('second')
'response'
>>> mock.assert_called_with('second')
```

Which approach you prefer is a matter of taste. The first approach is actually
a line shorter but maybe the second approach is more readable.

## Nesting Patches¶

Using patch as a context manager is nice, but if you do multiple patches you
can end up with nested with statements indenting further and further to the
right:

```
>>> class MyTest(TestCase):
...
...     def test_foo(self):
...         with patch('mymodule.Foo') as mock_foo:
...             with patch('mymodule.Bar') as mock_bar:
...                 with patch('mymodule.Spam') as mock_spam:
...                     assert mymodule.Foo is mock_foo
...                     assert mymodule.Bar is mock_bar
...                     assert mymodule.Spam is mock_spam
...
>>> original = mymodule.Foo
>>> MyTest('test_foo').test_foo()
>>> assert mymodule.Foo is original
```

With unittest2 cleanup functions and the *patch methods: start and stop* we can
achieve the same effect without the nested indentation. A simple helper
method, create\_patch, puts the patch in place and returns the created mock
for us:

```
>>> class MyTest(TestCase):
...
...     def create_patch(self, name):
...         patcher = patch(name)
...         thing = patcher.start()
...         self.addCleanup(patcher.stop)
...         return thing
...
...     def test_foo(self):
...         mock_foo = self.create_patch('mymodule.Foo')
...         mock_bar = self.create_patch('mymodule.Bar')
...         mock_spam = self.create_patch('mymodule.Spam')
...
...         assert mymodule.Foo is mock_foo
...         assert mymodule.Bar is mock_bar
...         assert mymodule.Spam is mock_spam
...
>>> original = mymodule.Foo
>>> MyTest('test_foo').run()
>>> assert mymodule.Foo is original
```

## Mocking a dictionary with MagicMock¶

You may want to mock a dictionary, or other container object, recording all
access to it whilst having it still behave like a dictionary.

We can do this with MagicMock, which will behave like a dictionary,
and using side\_effect to delegate dictionary access to a real
underlying dictionary that is under our control.

When the \_\_getitem\_\_ and \_\_setitem\_\_ methods of our MagicMock are called
(normal dictionary access) then side\_effect is called with the key (and in
the case of \_\_setitem\_\_ the value too). We can also control what is returned.

After the MagicMock has been used we can use attributes like
call\_args\_list to assert about how the dictionary was used:

```
>>> my_dict = {'a': 1, 'b': 2, 'c': 3}
>>> def getitem(name):
...      return my_dict[name]
...
>>> def setitem(name, val):
...     my_dict[name] = val
...
>>> mock = MagicMock()
>>> mock.__getitem__.side_effect = getitem
>>> mock.__setitem__.side_effect = setitem
```

Note

An alternative to using MagicMock is to use Mock and *only* provide
the magic methods you specifically want:

```
>>> mock = Mock()
>>> mock.__setitem__ = Mock(side_effect=getitem)
>>> mock.__getitem__ = Mock(side_effect=setitem)
```

A *third* option is to use MagicMock but passing in dict as the spec
(or spec\_set) argument so that the MagicMock created only has
dictionary magic methods available:

```
>>> mock = MagicMock(spec_set=dict)
>>> mock.__getitem__.side_effect = getitem
>>> mock.__setitem__.side_effect = setitem
```

With these side effect functions in place, the mock will behave like a normal
dictionary but recording the access. It even raises a KeyError if you try
to access a key that doesn’t exist.

```
>>> mock['a']
1
>>> mock['c']
3
>>> mock['d']
Traceback (most recent call last):
    ...
KeyError: 'd'
>>> mock['b'] = 'fish'
>>> mock['d'] = 'eggs'
>>> mock['b']
'fish'
>>> mock['d']
'eggs'
```

After it has been used you can make assertions about the access using the normal
mock methods and attributes:

```
>>> mock.__getitem__.call_args_list
[call('a'), call('c'), call('d'), call('b'), call('d')]
>>> mock.__setitem__.call_args_list
[call('b', 'fish'), call('d', 'eggs')]
>>> my_dict
{'a': 1, 'c': 3, 'b': 'fish', 'd': 'eggs'}
```

## Mock subclasses and their attributes¶

There are various reasons why you might want to subclass Mock. One reason
might be to add helper methods. Here’s a silly example:

```
>>> class MyMock(MagicMock):
...     def has_been_called(self):
...         return self.called
...
>>> mymock = MyMock(return_value=None)
>>> mymock
<MyMock id='...'>
>>> mymock.has_been_called()
False
>>> mymock()
>>> mymock.has_been_called()
True
```

The standard behaviour for Mock instances is that attributes and the return
value mocks are of the same type as the mock they are accessed on. This ensures
that Mock attributes are Mocks and MagicMock attributes are MagicMocks
[2]. So if you’re subclassing to add helper methods then they’ll also be
available on the attributes and return value mock of instances of your
subclass.

```
>>> mymock.foo
<MyMock name='mock.foo' id='...'>
>>> mymock.foo.has_been_called()
False
>>> mymock.foo()
<MyMock name='mock.foo()' id='...'>
>>> mymock.foo.has_been_called()
True
```

Sometimes this is inconvenient. For example, one user is subclassing mock to
created a Twisted adaptor.
Having this applied to attributes too actually causes errors.

Mock (in all its flavours) uses a method called \_get\_child\_mock to create
these “sub-mocks” for attributes and return values. You can prevent your
subclass being used for attributes by overriding this method. The signature is
that it takes arbitrary keyword arguments (\*\*kwargs) which are then passed
onto the mock constructor:

```
>>> class Subclass(MagicMock):
...     def _get_child_mock(self, **kwargs):
...         return MagicMock(**kwargs)
...
>>> mymock = Subclass()
>>> mymock.foo
<MagicMock name='mock.foo' id='...'>
>>> assert isinstance(mymock, Subclass)
>>> assert not isinstance(mymock.foo, Subclass)
>>> assert not isinstance(mymock(), Subclass)
```

|  |  |
| --- | --- |
| [2] | An exception to this rule are the non-callable mocks. Attributes use the callable variant because otherwise non-callable mocks couldn’t have callable methods. |

## Mocking imports with patch.dict¶

One situation where mocking can be hard is where you have a local import inside
a function. These are harder to mock because they aren’t using an object from
the module namespace that we can patch out.

Generally local imports are to be avoided. They are sometimes done to prevent
circular dependencies, for which there is *usually* a much better way to solve
the problem (refactor the code) or to prevent “up front costs” by delaying the
import. This can also be solved in better ways than an unconditional local
import (store the module as a class or module attribute and only do the import
on first use).

That aside there is a way to use mock to affect the results of an import.
Importing fetches an *object* from the sys.modules dictionary. Note that it
fetches an *object*, which need not be a module. Importing a module for the
first time results in a module object being put in sys.modules, so usually
when you import something you get a module back. This need not be the case
however.

This means you can use patch.dict() to *temporarily* put a mock in place
in sys.modules. Any imports whilst this patch is active will fetch the mock.
When the patch is complete (the decorated function exits, the with statement
body is complete or patcher.stop() is called) then whatever was there
previously will be restored safely.

Here’s an example that mocks out the ‘fooble’ module.

```
>>> mock = Mock()
>>> with patch.dict('sys.modules', {'fooble': mock}):
...    import fooble
...    fooble.blob()
...
<Mock name='mock.blob()' id='...'>
>>> assert 'fooble' not in sys.modules
>>> mock.blob.assert_called_once_with()
```

As you can see the import fooble succeeds, but on exit there is no ‘fooble’
left in sys.modules.

This also works for the from module import name form:

```
>>> mock = Mock()
>>> with patch.dict('sys.modules', {'fooble': mock}):
...    from fooble import blob
...    blob.blip()
...
<Mock name='mock.blob.blip()' id='...'>
>>> mock.blob.blip.assert_called_once_with()
```

With slightly more work you can also mock package imports:

```
>>> mock = Mock()
>>> modules = {'package': mock, 'package.module': mock.module}
>>> with patch.dict('sys.modules', modules):
...    from package.module import fooble
...    fooble()
...
<Mock name='mock.module.fooble()' id='...'>
>>> mock.module.fooble.assert_called_once_with()
```

## Tracking order of calls and less verbose call assertions¶

The Mock class allows you to track the *order* of method calls on
your mock objects through the method\_calls attribute. This
doesn’t allow you to track the order of calls between separate mock objects,
however we can use mock\_calls to achieve the same effect.

Because mocks track calls to child mocks in mock\_calls, and accessing an
arbitrary attribute of a mock creates a child mock, we can create our separate
mocks from a parent one. Calls to those child mock will then all be recorded,
in order, in the mock\_calls of the parent:

```
>>> manager = Mock()
>>> mock_foo = manager.foo
>>> mock_bar = manager.bar

>>> mock_foo.something()
<Mock name='mock.foo.something()' id='...'>
>>> mock_bar.other.thing()
<Mock name='mock.bar.other.thing()' id='...'>

>>> manager.mock_calls
[call.foo.something(), call.bar.other.thing()]
```

We can then assert about the calls, including the order, by comparing with
the mock\_calls attribute on the manager mock:

```
>>> expected_calls = [call.foo.something(), call.bar.other.thing()]
>>> manager.mock_calls == expected_calls
True
```

If patch is creating, and putting in place, your mocks then you can attach
them to a manager mock using the attach\_mock() method. After
attaching calls will be recorded in mock\_calls of the manager.

```
>>> manager = MagicMock()
>>> with patch('mymodule.Class1') as MockClass1:
...     with patch('mymodule.Class2') as MockClass2:
...         manager.attach_mock(MockClass1, 'MockClass1')
...         manager.attach_mock(MockClass2, 'MockClass2')
...         MockClass1().foo()
...         MockClass2().bar()
...
<MagicMock name='mock.MockClass1().foo()' id='...'>
<MagicMock name='mock.MockClass2().bar()' id='...'>
>>> manager.mock_calls
[call.MockClass1(),
 call.MockClass1().foo(),
 call.MockClass2(),
 call.MockClass2().bar()]
```

If many calls have been made, but you’re only interested in a particular
sequence of them then an alternative is to use the
assert\_has\_calls() method. This takes a list of calls (constructed
with the call object). If that sequence of calls are in
mock\_calls then the assert succeeds.

```
>>> m = MagicMock()
>>> m().foo().bar().baz()
<MagicMock name='mock().foo().bar().baz()' id='...'>
>>> m.one().two().three()
<MagicMock name='mock.one().two().three()' id='...'>
>>> calls = call.one().two().three().call_list()
>>> m.assert_has_calls(calls)
```

Even though the chained call m.one().two().three() aren’t the only calls that
have been made to the mock, the assert still succeeds.

Sometimes a mock may have several calls made to it, and you are only interested
in asserting about *some* of those calls. You may not even care about the
order. In this case you can pass any\_order=True to assert\_has\_calls:

```
>>> m = MagicMock()
>>> m(1), m.two(2, 3), m.seven(7), m.fifty('50')
(...)
>>> calls = [call.fifty('50'), call(1), call.seven(7)]
>>> m.assert_has_calls(calls, any_order=True)
```

## More complex argument matching¶

Using the same basic concept as ANY we can implement matchers to do more
complex assertions on objects used as arguments to mocks.

Suppose we expect some object to be passed to a mock that by default
compares equal based on object identity (which is the Python default for user
defined classes). To use assert\_called\_with() we would need to pass
in the exact same object. If we are only interested in some of the attributes
of this object then we can create a matcher that will check these attributes
for us.

You can see in this example how a ‘standard’ call to assert\_called\_with isn’t
sufficient:

```
>>> class Foo(object):
...     def __init__(self, a, b):
...         self.a, self.b = a, b
...
>>> mock = Mock(return_value=None)
>>> mock(Foo(1, 2))
>>> mock.assert_called_with(Foo(1, 2))
Traceback (most recent call last):
    ...
AssertionError: Expected: call(<__main__.Foo object at 0x...>)
Actual call: call(<__main__.Foo object at 0x...>)
```

A comparison function for our Foo class might look something like this:

```
>>> def compare(self, other):
...     if not type(self) == type(other):
...         return False
...     if self.a != other.a:
...         return False
...     if self.b != other.b:
...         return False
...     return True
...
```

And a matcher object that can use comparison functions like this for its
equality operation would look something like this:

```
>>> class Matcher(object):
...     def __init__(self, compare, some_obj):
...         self.compare = compare
...         self.some_obj = some_obj
...     def __eq__(self, other):
...         return self.compare(self.some_obj, other)
...
```

Putting all this together:

```
>>> match_foo = Matcher(compare, Foo(1, 2))
>>> mock.assert_called_with(match_foo)
```

The Matcher is instantiated with our compare function and the Foo object
we want to compare against. In assert\_called\_with the Matcher equality
method will be called, which compares the object the mock was called with
against the one we created our matcher with. If they match then
assert\_called\_with passes, and if they don’t an AssertionError is raised:

```
>>> match_wrong = Matcher(compare, Foo(3, 4))
>>> mock.assert_called_with(match_wrong)
Traceback (most recent call last):
    ...
AssertionError: Expected: ((<Matcher object at 0x...>,), {})
Called with: ((<Foo object at 0x...>,), {})
```

With a bit of tweaking you could have the comparison function raise the
AssertionError directly and provide a more useful failure message.

As of version 1.5, the Python testing library PyHamcrest provides similar functionality,
that may be useful here, in the form of its equality matcher
(hamcrest.library.integration.match\_equality).

## Less verbose configuration of mock objects¶

This recipe, for easier configuration of mock objects, is now part of Mock.
See the configure\_mock() method.

## Matching any argument in assertions¶

This example is now built in to mock. See ANY.

## Mocking Properties¶

This example is now built in to mock. See PropertyMock.

## Mocking open¶

This example is now built in to mock. See mock\_open().

## Mocks without some attributes¶

This example is now built in to mock. See *Deleting Attributes*.

### Table Of Contents

- Further Examples
  - Mocking chained calls
  - Partial mocking
  - Mocking a Generator Method
  - Applying the same patch to every test method
  - Mocking Unbound Methods
  - Checking multiple calls with mock
  - Coping with mutable arguments
  - Raising exceptions on attribute access
  - Multiple calls with different effects
  - Nesting Patches
  - Mocking a dictionary with MagicMock
  - Mock subclasses and their attributes
  - Mocking imports with patch.dict
  - Tracking order of calls and less verbose call assertions
  - More complex argument matching
  - Less verbose configuration of mock objects
  - Matching any argument in assertions
  - Mocking Properties
  - Mocking open
  - Mocks without some attributes

#### Previous topic

Getting Started with Mock

#### Next topic

Mock Library Comparison

### This Page

- Show Source

### Quick search


Enter search terms or a module, class or function name.

### Navigation

- index
- next |
- previous |
- Mock 1.0.1 documentation »

© Copyright 2007-2012, Michael Foord & the mock team.
Last updated on Nov 05, 2012.
Created using Sphinx 1.1.3.
